# Boehringer Ingelheim and Eli Lilly and Company announces trategic alliance to bring new diabetes treatments to patients worldwide

**Published:** 2011

- Major diabetes care agreement centres on four pipeline compounds representing several of the largest and most promising product classes.
- Companies will jointly develop and commercialise a pipeline of oral diabetes agents and basal insulin analogues. The alliance also includes the option to co-develop and co-commercialise an anti-TGF-beta monoclonal antibody.
- Boehringer Ingelheim’s innovative late-stage diabetes pipeline drives its expansion into a new therapeutic area, supplemented by two basal insulin analogues, currently under development from Lilly.
- Agreement furthers Lilly’s commitment to offer one of the broadest portfolios in diabetes care and provide, together with Boehringer Ingelheim, more options for people with diabetes, their healthcare providers and payers.
- Boehringer Ingelheim hosted a media event and Lilly hosted an investor conference call to discuss the content and benefits of the alliance.

Boehringer Ingelheim (Germany and Indianapolis, USA) and Eli Lilly and Company (NYSE: LLY) recently announced a global agreement to jointly develop and commercialise a portfolio of diabetes compounds currently in midand late-stage development. Included are Boehringer Ingelheim’s two oral diabetes agents, linagliptin and BI10773, and Lilly’s two basal insulin analogues, LY2605541 and LY2963016, as well as the option to co-develop and co-commercialise Lilly’s anti-TGF-beta monoclonal antibody.

Linagliptin is a dipeptidyl peptidase- 4 (DPP-4) inhibitor discovered by Boehringer Ingelheim and being developed as an oral once-daily tablet for the treatment of type 2 diabetes. It is currently under regulatory review in the USA, Europe and Japan. Boehringer Ingelheim’s BI10773, a sodium-dependent glucose co-transporter-2 (SGLT-2) inhibitor, began enrolment in phase III clinical trials last year. It belongs to a new, emerging class of diabetes compounds that block tubular re-absorption of glucose in the kidney. Currently there are no SGLT-2 inhibitors approved for use.

Lilly’s two basal insulin analogue candidates are expected to enter phase III clinical testing in 2011. These are LY2605541, a structurally novel basal insulin analogue, and LY2963016, a new insulin glargine product. The agreement also includes an option for Boehringer Ingelheim to co-develop and co-commercialise another Lilly diabetes molecule, an anti-TGF-beta monoclonal antibody, which is currently in phase II of clinical testing in patients with diabetes and chronic kidney disease.

The alliance will leverage the collective scientific expertise and business capabilities of two leading research-driven pharmaceutical companies to address patient needs arising from the growing global diabetes epidemic.

‘We are very excited about this new and extensive alliance with Boehringer Ingelheim, with whom we have partnered successfully in the past’, said John C Lechleiter, PhD, Lilly chairman and chief executive officer. ‘Working together, we will comprise one of the most robust diabetes pipelines in the pharmaceutical industry. For Lilly, this alliance expands our range of offerings for people with diabetes, strengthens our diabetes care capabilities and offers the prospect of near-term revenue opportunities as we address the upcoming loss of patent exclusivity for several of our products.’

‘Boehringer Ingelheim and Lilly have agreed to form a strategic alliance in diabetes at a time point when we at Boehringer Ingelheim are entering another new therapeutic area with innovative compounds from our own research and development. This cooperation will give Boehringer Ingelheim and Lilly the combined benefits of Lilly’s expertise in the diabetes market and two basal insulin analogues, as well as Boehringer Ingelheim’s rich and innovative late-stage pipeline’, said Prof Andreas Barner, chairman of the Board of Managing Directors of Boehringer Ingelheim.

For further information please contact: Ms Sue Thomas (Medical Information Manager) on +27 11 348 2514 or Dr Kevin Ho (Medical Director) on +27 11 348 2517.
